# Combination of ferulic acid, ligustrazine and tetrahydropalmatine inhibits invasion and metastasis through MMP/TIMP signaling in endometriosis

**DOI:** 10.7717/peerj.11664

**Published:** 2021-06-28

**Authors:** Yi Tan, Chengling Zhang, Ying Zhang, Xueshan Dai, Qinghua Wei, Jiahui Wei, Pingli Xu, Yi Chen

**Affiliations:** 1 College of Pharmaceutical Sciences & Chinese Medicine, Southwest University, Chongqing, China; 2 Chongqing Key Laboratory of New Drug Screening from Traditional Chinese Medicine, Chongqing, China; 3 Pharmacology of Chinese Materia Medica–the Key Discipline Constructed by the State Administration of Traditional Chinese Medicine, Chongqing, China; 4 National Demonstration Center for Experimental Pharmacy Education (Southwest University), Chongqing, China; 5 Chongqing Hospital of Traditional Chinese Medicine, Chongqing, China

**Keywords:** Ferulic acid, Ligustrazine, Tetrahydropalmatine, Endometriosis, Invasion and metastasis, MMP/TIMP signaling

## Abstract

**Background:**

The design of the combination of ferulic acid, ligustrazine and tetrahydropalmatine (FLT) is inspired by the Chinese herbal prescription Foshou San. Previous work has shown that FLT inhibited endometriosis growth in rat autograft models. However, the mechanism behind this is unclear. MMP/TIMP signaling is considered as the vital pathway of metastasis and invasion in endometriosis. In this study, we aim to disclose effects of FLT on MMP/TIMP signaling in invasion and metastasis during endometrial cells and xenograft endometriosis.

**Methods:**

In vivo, effect of FLT on endometriosis was evaluated in a xenogeneic mice model. In vitro, cell viability assay was performed with an IC50 measurement of FLT in hEM15A and HEC1-B cells. The effect of FLT on invasion and metastasis was analyzed in scratch wound and transwell assay. Gene and protein expression of MMP/TIMP signaling were detected by qPCR and Western blotting.

**Results:**

In xenograft endometriosis, FLT reduced ectopic volume without effect on weight. FLT inhibitory effects on cell growth exhibited a dose-dependent manner in hEM15A and HEC1-B cells. IC50s of FLT in hEM15A cells were 839.30 ± 121.11 or 483.53 ±156.91 μg·ml^−1^ after the treatment for 24 or 48 h, respectively. In HEC1-B cells, IC50 values of 24 or 48 h were 625.20 ± 59.52 or 250.30 ± 68.12 μg·ml^−1^. In addition, FLT significantly inhibited invasion and metastasis in scratch wound and transwell assay. Furthermore, FLT inactivated MMP/TIMP signaling with decreasing expression of MMP-2/9, and an enhancing expression of TIMP-1.

**Conclusions:**

MMP/TIMP inactivation is a reasonable explanation for the inhibition of FLT on invasion and metastasis in endometriosis. This result reveals a potential mechanism on the role of FLT in endometriosis and may benefit for its further application.

## Introduction

Endometriosis (EMS) is a disease caused by active endometrial cells growing outside the endometrium. Although EMS pathogenesis is unclear, endometrial implantation is essential in menstrual effluent by menstrual reflux hypothesis. This pathological step mainly contains invasion and metastasis (Chui, Wang & Shih, 2017; Mihailovici et al., 2017). Furthermore, vital mechanism of invasion and metastasis includes imbalance between matrix metalloproteinases (MMPs) and tissue inhibitors of MMPs (TIMPs). MMP-2 and MMP-9 promote invasion and metastasis through matrix degradation. On the contrary, TIMP-1 shows inhibitory effects on MMPs (Herszenyi et al., 2012).

EMS belongs to blood stasis syndromes, which is treated by activating blood and dissolving stasis in traditional Chinese medicine (Chen et al., 2018; Liao, 2000). The famous gynecological prescription Foshou san is performed in blood stasis syndromes, including EMS (Bai et al., 2014). FLT is a new mixture derived from Foshou san, composed of ferulic acid, ligustrazine and tetrahydropalmatine. In autograft EMS, potential roles of FLT in anti-inflammation, inhibiting epithelial mesenchymal transition, and inducing apoptosis have been indicated (Chen et al., 2018; Tang et al., 2014; Wei et al., 2019). But it is still unknown whether FLT has a similar effect in allograft EMS and endometrial cells.

In this paper, therapeutic effect of FLT on endometriosis was investigated in allograft mice model. IC50 FLT on hEM15A and HEC1-B cells were measured in vitro. Afterwards, anti-metastasis was detected by scratch wound and transwell assays. Regulations of MMP/TIMP signaling were investigated by FLT in vitro and in vivo.

## Materials & methods

### Animals and chemicals

Female C3H mice, aged 8 ± 1 weeks, weighing 19 ± 1 g, were purchased from Beijing Vital River Laboratory Animal Technology Co., Ltd. (Certification no. SCXK [Jing]2016-0006) (Beijing, China). Female nude mice, aged 8 ± 1 weeks, weighing 19 ± 1 g were purchased from Hunan Silaike Jingda Laboratory Animal Co., Ltd. (Certification no. SCXK [Xiang]2016-0002) (Hunan, China). The mice were sheltered at a Ta of 20 ± 2 °C and 12 h light/dark cycle. They were supplied with enough food and water in Experimental Center of Southwest University. This study was executed in rigorous accordance with the recommendations in the Guidelines for the Care and Use of Laboratory Animals of Southwest University. This study was approved by the Ethics Committee of the School of Pharmacy, Southwest University (Approval no. 201702). To minimize suffering, anaesthesia and other necessary efforts were performed. A total of 350 or 1,750 mg·kg^−1^ chloral hydrate was administered intraperitoneally in experimental or sacrificed procedure separately. All animals were sacrificed at human endpoint.

Ferulic acid, ligustrazine and tetrahydropalmatine were purchased from Nanjing Zelang Medical Technology Co., Ltd (Nanjing, China), with the purity of 99.8, 99.3, 98.1% respectively. In vitro, the compounds dissolved in DMSO were mixed at the ratio of 1:0.5:0.3 for cells treatment. In vivo, three compounds mixed with the same ratio in 0.5% CMC-Na for mice treatment. Gestrinone was used as a positive drug provided by Zizhu Pharmaceutical Co., Ltd. Beijing, China.

### Allograft EMS model and treatment

Allograft EMS mice model was established following previous protocols (Attaman et al., 2014; Cheng et al., 2011). C3H and nude mice were anaesthetized before operation. Bilateral uterus of estrus C3H mice were cut into 4 mm^2^ blocks and transplanted into subcutaneous abdomen of 40 nude mice. After operation, 2 mg·kg^−1^ estradiol benzoate was intramuscularly administrated in nude mice once every 5 days. Ectopic volume was calculated by vernier caliper after 28 days with a formula (0.52 × length × width × height). Endometrial ectopic explants were larger than 4.5 mm^3^ with surface blood, indicating that EMS models were successful established. Next, 30 EMS mice randomly were divided into five groups based on random number list, 6 mice in each group. Then the mice were named after the EMS, FLT, and gestrinone groups and treated with 0.5% CMC-Na, 90, 180, 360 mg·kg^−1^ FLT and 0.05 mg·kg^−1^ gestrinone, respectively. Another 6 female C3H mice without transplantation were administered with 0.5% CMC-Na as control group. During 28 days’ treatment, weight of nude mice was measured every 7 days. After administration for 28 days, ectopic volumes were measured again.

### Cell culture

Human EMS-derived eutopic endometrium stromal cells hEM15A presented by Professor Xiaohong Chang of Peking University People’s Hospital (Beijing, China). The endometrial adenocarcinoma cells HEC1-B and human kidney 293T cells were purchased from the Chinese Centre for Type Cultures Collections (CCTCC, Wuhan, China). hEM15A, HEC1-B or 293T cells were cultured in DMEM/F12 or MEM medium (Gibco, Grand Island, NY, USA) with 10% FBS (Hyclone, Shanghai, China) at 37 °C with 5% CO_2_.

### Cell viability assay

hEM15A, HEC1-B and 293T cells were seeded at 6-8 × 10^3^, 8–10 × 10^3^, 8–10 × 10^3^ cells/well in 96-well plates respectively. hEM15A cells were treated with FLT (0, 156, 313, 625, 1,250 and 2,500 μg·ml^−1^), and HEC1-B cells were cultured with FLT (0, 15, 46, 139, 417 and 1,250 μg·ml^−1^) for 24 or 48 h. Meanwhile, 0, 840, 1,680 and 3,360 μg·ml^−1^ FLT were used in 293T cells for 24 or 48 h. After treatment, cells were incubated with MTT solution for 4 h. Then cells were measured in a microplate reader after dissolved in DMSO. IC50 values of hEM15A, HEC1-B cells were calculated by GraphPad Prism5 Software (5.01 version, La Jolla, CA, USA).

### Scratch wound assay

hEM15A and HEC1-B cells were put in 24-well plate at a density of 6–8 × 10^4^, 8–10 × 10^4^ cells/well. When cell confluence reached approximately 80%, the middle of well was vertically scratched by 1 ml pipette tip. Using FLT, endometrial cells were observed and photographed under 50× magnification at 0, 6, 12, 24 and 48 h. Scratched area was calculated using Image pro plus 6.0 software (Media Cybernetics, Silver Spring, southeastern Montgomery County, MD, USA). Scratch closure rate = (average scratch area at 0 h − average scratch area at each time point)/(average scratch area at 0 h) × 100%.

### Transwell assay

Transwell inserts (Corning, New York, NY, USA) without matrigel were put into 24-well plate. A total of 3 × 10^4^ hEM15A or HEC1-B cells/well were placed into upper chamber with serum-free FLT medium. A total of 5% FBS medium without FLT were added to lower chamber. After incubation at 37 °C for 6, 12, 24 and 48 h, surface cells on upper chamber were swiped by cotton swab. Migrated cells in bottom were fixed with paraformaldehyde, and then stained with crystal violet. Migratory cell numbers were counted in 5 random fields at 100× magnification.

### RNA isolation and qPCR

Total RNA was extracted using TRIzol reagent (Invitrogen, Carlsbad, CA, USA) and then reversely transcribed into cDNA with PrimeScript™ Reagent Kit (Takara, China). Real-time PCR was conducted in CFX96 Real-Time System (Bio-Rad, Hercules, CA, USA) and fluorescent quantification was detected with SYBR™ Green Master Mix (Thermo Fisher Scientific, Waltham, MA, USA). Primer of mice and human were synthesized by Dingguo Changsheng Biotechnology (Beijing, China). Relative mRNA expression was calculated by 2−∆∆CT method using primer sequences (Table 1), which. GAPDH gene as the control reference.

**Table 1:** Primer sequences of RT-qPCR.

Species	Primer name	Sequences (5′-3′)
Mouse	MMP-2-F	CCCTCAAGAAGATGCAGAAGTTC
	MMP-2-R	ATCTTGGCTTCCGCATGGT
	MMP-9-F	ACCAAGGGTACAGCCTGTTCCT
	MMP-9-R	GGTAGCTATACAGCGGGTACATGA
	TIMP-1-F	CTTGGTTCCCTGGCGTACTC
	TIMP-1-R	ACCTGATCCGTCCACAAACAG
	GAPDH-F	CCTGGAGAAACCTGCCAAGTAT
	GAPDH-R	GGTCCTCAGTGTAGCCCAAGAT
Human	MMP-2-F	GGCCCTGTCACTCCTGAGAT
	MMP-2-R	GGCATCCAGGTTATCGGGGA
	MMP-9-F	TGGACGATGCCTGCAACGTG
	MMP-9-R	GTCGTGCGTGTCCAAAGGCA
	TIMP-1-F	CAATTCCGACCTCGTCATCAG
	TIMP-1-R	CTTGGAACCCTTTATACATCTTGG
	GAPDH-F	AATGGGCAGCCGTTAGGAAA
	GAPDH-R	GCCCAATACGACCAAATCAGAG

### Western blot analysis

Total Proteins of tissues and cells were extracted by RIPA lysis buffer with protease inhibitors on ice. Equal amounts of protein were separated by SDS-PAGE and transferred onto poplvinylidene fluoride membrane (Millipore, USA). The membranes were incubated overnight at 4 °C with primary rabbit anti-MMP-2 and rabbit anti-MMP-9 (1:100 dilution; Boster Biological Technology, Wuhan, China), rabbit anti-TIMP-1 (1:300 dilution; Proteintech Biotechnology, Wuhan, China), rabbit anti-β-actin (1:5,000 dilution; Proteintech Biotechnology, Wuhan, China). After washing with TBST, membranes were incubated with HRP-labeled goat anti-rabbit secondary antibody (1:1,000 dilution; Multi Sciences, Hangzhou, China). Chemiluminescent signals were captured analyzed with the Tanon 5200 imaging system (Tanon, Shanghai, China). β-actin was used as an internal control.

### Statistical analysis

All data were presented as the mean ± SD. They were analyzed by one-way ANOVA test in SPSS 21.0 software. *P* < 0.05 was considered statistically significant. Yi Chen was aware of the group allocation at the different stages of the experiment.

## Results

### Inhibition of ectopic endometrium volume using FLT

**Figure 1:**
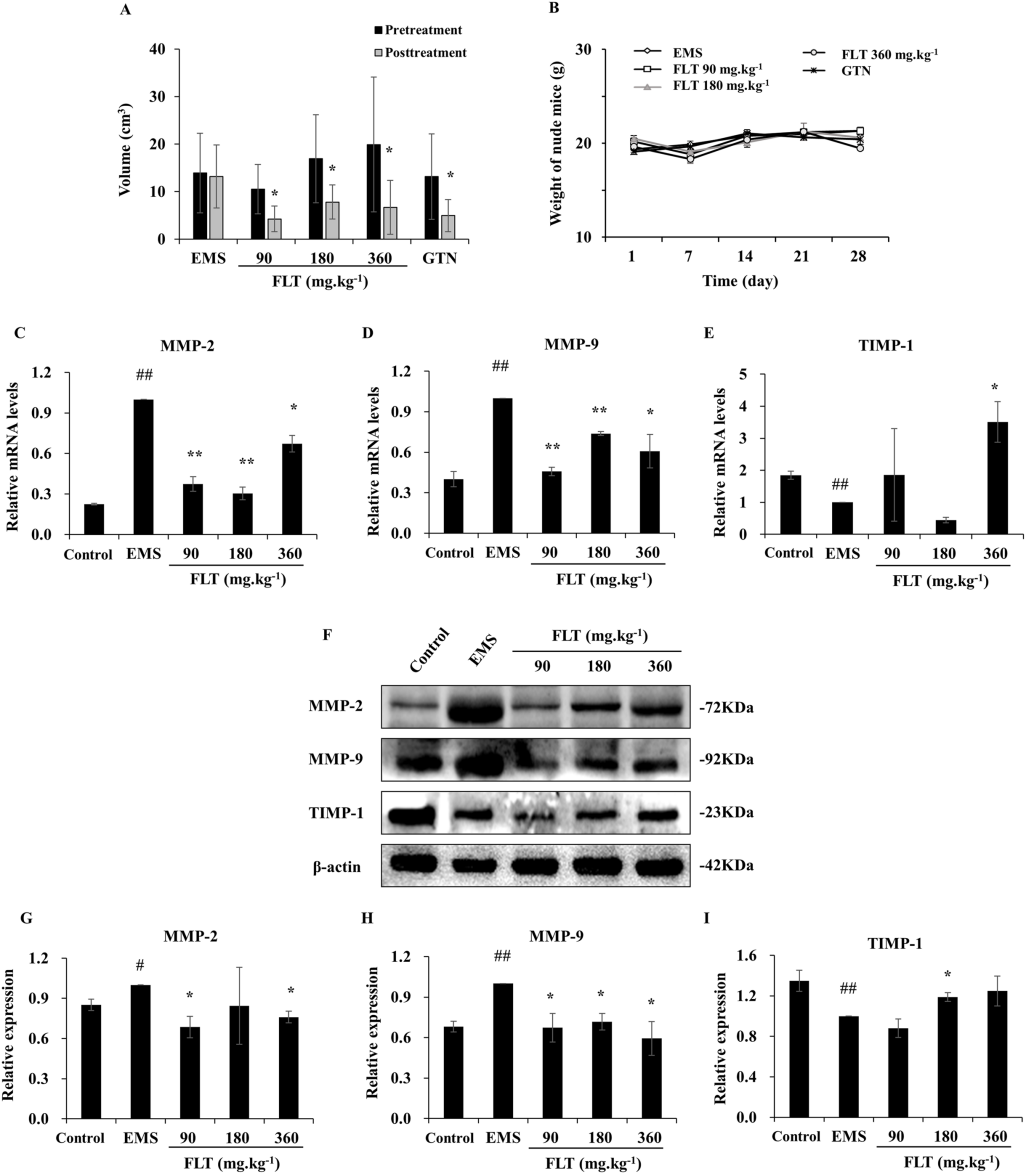
Regulation of FLT on xenograft EMS. (A) Ectopic volume was detected by vernier caliper in xenograft EMS model. GTN was performed as positive control. (B) Weight of nude mice was measured every 7 days during 28 days’ treatment. *P < 0.05 to pretreatment. Columns, mean (*n* = 6). (C–E) Ectopic endometrium of EMS and FLT groups were collected for RNA isolation. Eutopic endometrium of C3H mice without transplantation were supplied as the control group. mRNA levels of MMP-2, MMP-9, and TIMP-1 were detected by qPCR in different groups. (F–I) Protein levels of MMP-2, MMP-9, and TIMP-1 were measured by western blotting, and the ratio of MMP-2, MMP-9, and TIMP-1 with β-actin were shown. #*P* < 0.05 to control, ##*P* < 0.01 to control, *P < 0.05 to EMS, **P < 0.01 to EMS. Columns, mean (*n* = 3). Bars, SD. EMS, endometriosis; FLT, ferulic acid, ligustrazine and tetrahydropalmatine; GTN, gestrinone.

A total of 28 days after transplantation, 30 of 40 nude mice were found allograft EMS in second laparotomy. The successful rate of model was 75%. Meantime, no remarkable difference of ectopic volume was found in all groups before administration. After treatment with FLT for 28 days, ectopic volume was detected and compared with pretreatment. In the EMS group, ectopic volume had no difference between posttreatment and pretreatment. There were significantly reduction in ectopic volume in 90, 180 and 360 mg·kg^−1^ FLT groups, respectively (*P* < 0.05). Gestrinone is currently applied in clinic as anti-progestagens steroid (Ferrero, Evangelisti & Barra, 2018), which was used as positive control in this study. Ectopic volume of endometrium tissue was also decreased in the gestrinone group (*P* < 0.05) (Fig. 1A). Moreover, weight of nude mice had no different between EMS, FLT and gestrinone groups (Fig. 1B). This suggests that FLT restrained EMS growth at all tested concentrations without obvious severe side effects.

### Adjustment of MMP/TIMP signaling by FLT in vivo

MMP/TIMP signaling is one of important pathway in metastasis and invasion (Herszenyi et al., 2012). While, interruption of metastasis and invasion lead to EMS amelioration (Chui, Wang & Shih, 2017). In this study, MMP/TIMP signaling was detected in ectopic tissues from EMS, FLT groups, and in eutopic tissues from control group of C3H mice without surgery. As evidenced by qRT-PCR, mRNA level of MMP-2 and MMP-9 were significantly higher while TIMP-1 was lower in the EMS group than those in the control group (*P* < 0.01). mRNA level of MMP-2 and MMP-9 were obviously downregulated by FLT. By contrast, TIMP-1 was upregulated in 360 mg·kg^−1^ FLT group than the EMS group (*P* < 0.05) (Figs. 1C–1E). The protein level was analyzed by Western blotting. MMP-2 and MMP-9 protein levels were remarkably raised while TIMP-1 protein level were decreased in the EMS group compared with those in the control group (*P* < 0.05). FLT obviously decreased the MMP-2 and MMP-9 protein expression. In contrast, FLT increased TIMP-1 protein level (*P* < 0.05) (Figs. 1F–1I).

### Effect of FLT on endometrial and 293T cells

**Figure 2:**
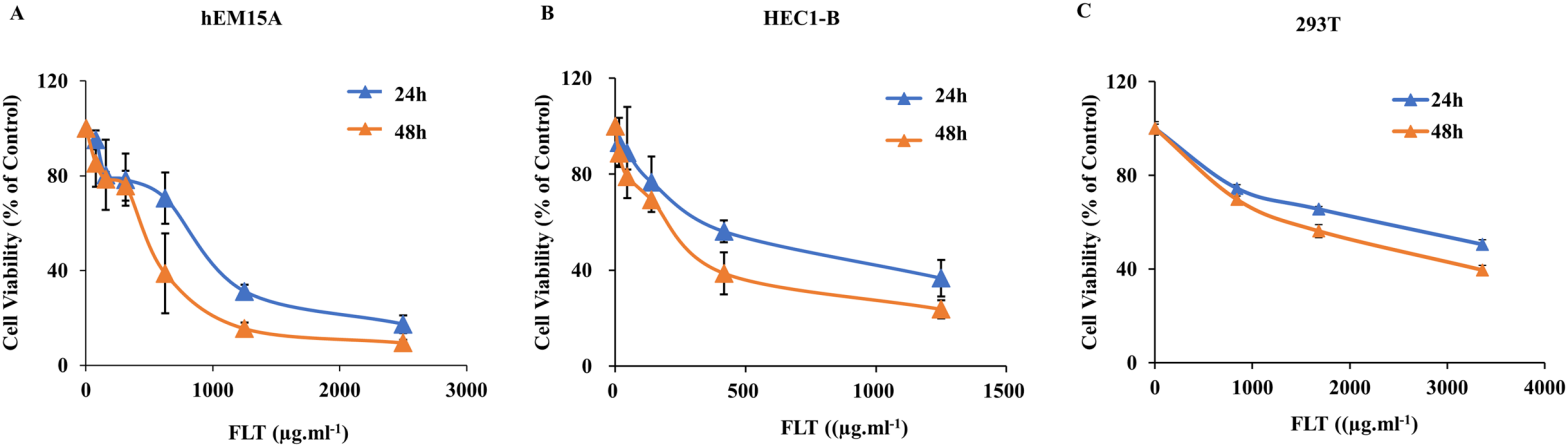
Effects of FLT on endometrial and 293T cells. hEM15A (A), HEC1-B (B) and 293T (C) cells were treated with various concentrations of FLT for 24 and 48 h, and then analyzed by MTT assay. Columns, mean (*n* = 3). Bars, SD. FLT, ferulic acid, ligustrazine and tetrahydropalmatine.

As noted above, FLT suppressed ectopic growth in vivo. But whether there was similar observation in vitro needs to be examined. Thus we measured the IC50s for the treatment of FLT on hEM15A and HEC1-B cells. 293T cells were performed for toxicity detection. In hEM15A cells, IC50 of FLT was 839.30 ± 121.11 or 483.53 ± 156.91 μg·ml^−1^ at 24 or 48 h respectively (Fig. 2A). In HEC1-B cells, IC50 value was 625.20 ± 59.52 or 250.30 ± 68.12 μg·ml^−1^ at 24 or 48 h respectively (Fig. 2B). Moreover, significant inhibitory effects of FLT were investigated with dose-dependent manner in both two cell lines. Notablely, HEC1-B cells was sensitive to FLT. The inhibitory effect of FLT on 293T cells was less than on hEM15A and HEC1-B cells with same concentration indicating a low toxicity in 293T (Fig. 2C).

### Constraint of migration and invasion by FLT in endometrial cell

**Figure 3:**
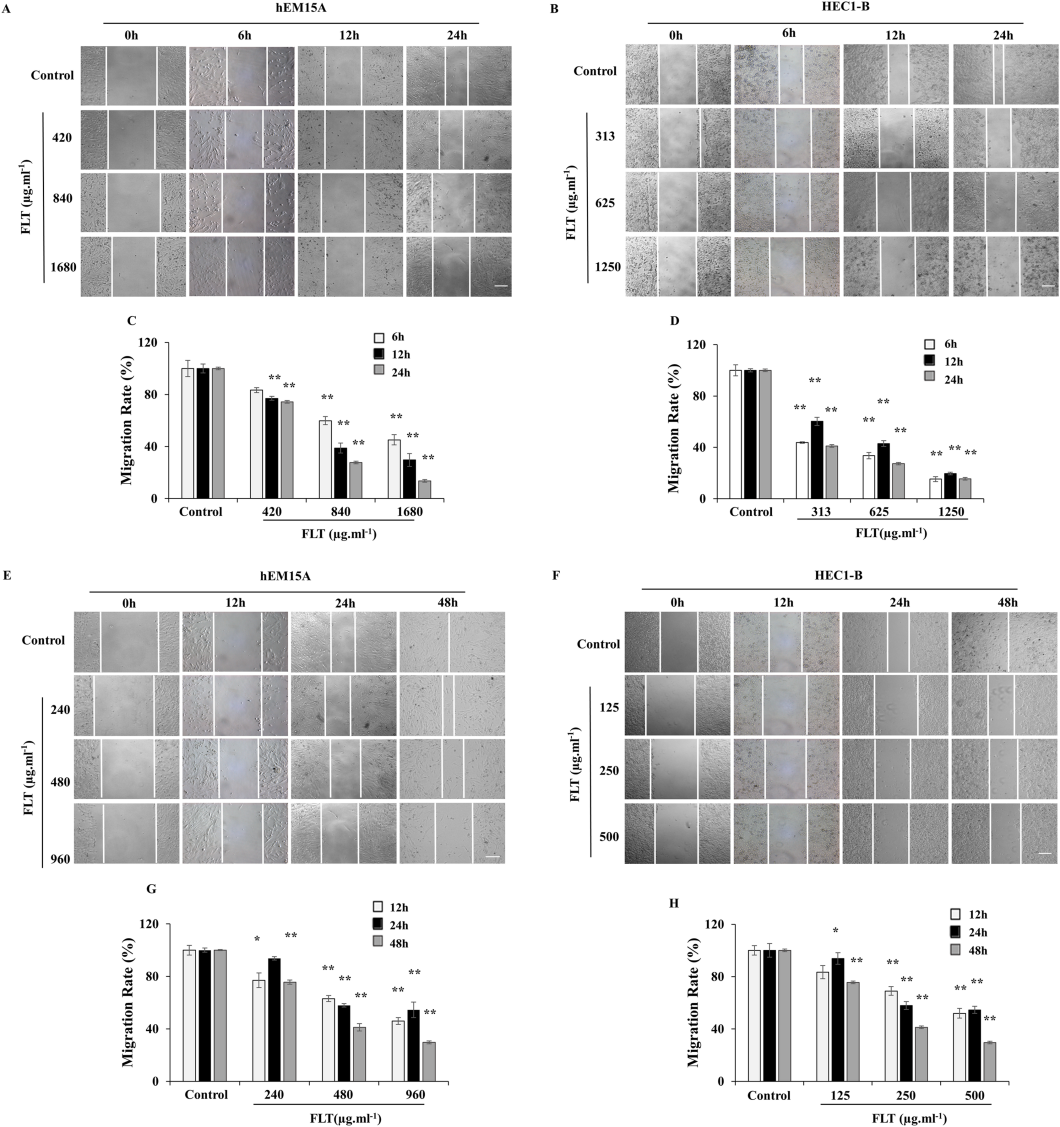
Migratory eﬀects of FLT on endometrial cells. hEM15A (A, C) and HEC1-B (B, D) cells were treated with FLT for 6, 12 and 24 h after scratch. hEM15A (E, G) and HEC1-B (F, H) cells were scratched by sterile pipette tip and treated with FLT for 12, 24 and 48 h. Photomicrographs showed representative wound scratches at diﬀerent time points after wounding. *P < 0.05 to control group, **P < 0.01 to control group. Columns, mean (*n* = 3). Bars, SD. Scale bar = 200 μm. FLT, ferulic acid, ligustrazine and tetrahydropalmatine.

Scratch wound assay is commonly used to detect the cell migration. IC50 concentration of FLT was performed as middle dose. Low or high dose was half or double of IC50 concentration. The endometrial cells were observed for 6, 12 and 24 h with IC50 24h FLT, or for 12, 24 and 48 h with IC50 48 h FLT. Wound closure of FLT were obviously decreased compared with control in hEM15A and HEC1-B cells (*P* < 0.01), except 420 μg·ml^−1^ FLT at 6h (Figs. 3A–3D). Meanwhile, FLT significantly resisted cells spread along the edge of wound scratch, with no effect in low dose of FLT at some time point (Figs. 3E–3H). This indicated that FLT markedly inhibited cell migration in scratch wound assay.

**Figure 4:**
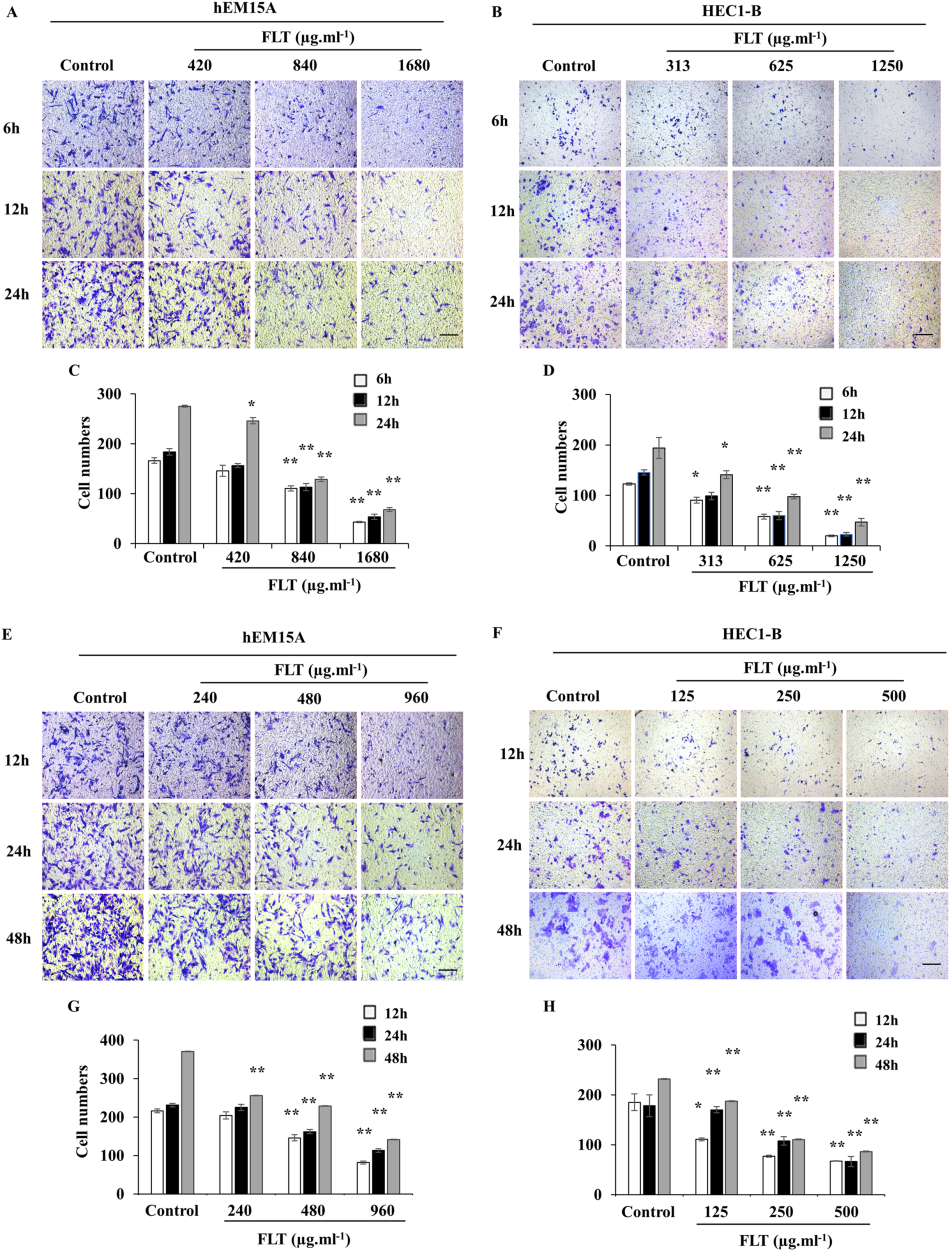
FLT restrained migration and invasion of endometrial cells. In transwell assay, hEM15A (A, C) and HEC1-B (B, D) cells were dealt with 24h IC50 concentration of FLT for 6, 12 and 24 h. hEM15A (E, G) and HEC1-B (F, H) cells were performed in transwell assay treated with IC50 48h FLT for 12, 24 and 48 h. The number of migrated cells was quantified in five random fields from three independent experiments. *P < 0.05 to control group, **P < 0.01 to control group. Columns, mean (*n* = 3). Bars, SD. Scale bar = 500 μm. FLT, ferulic acid, ligustrazine and tetrahydropalmatine.

Transwell assay was performed to measure FLT effects on cell migration and invasion. Maintain identical time and concentration of FLT were applied as previous scratch wound experiment. FLT significantly reduced migratory cells numbers vs control group in hEM15A (Figs. 4A, 4C, 4E, 4G) and HEC1-B cells (Figs. 4B, 4D, 4F, 4H). This suggested that FLT obviously suppressed cell migration and invasion in transwell assay.

### Regulation of FLT on MMP/TIMP signaling in vitro

**Figure 5:**
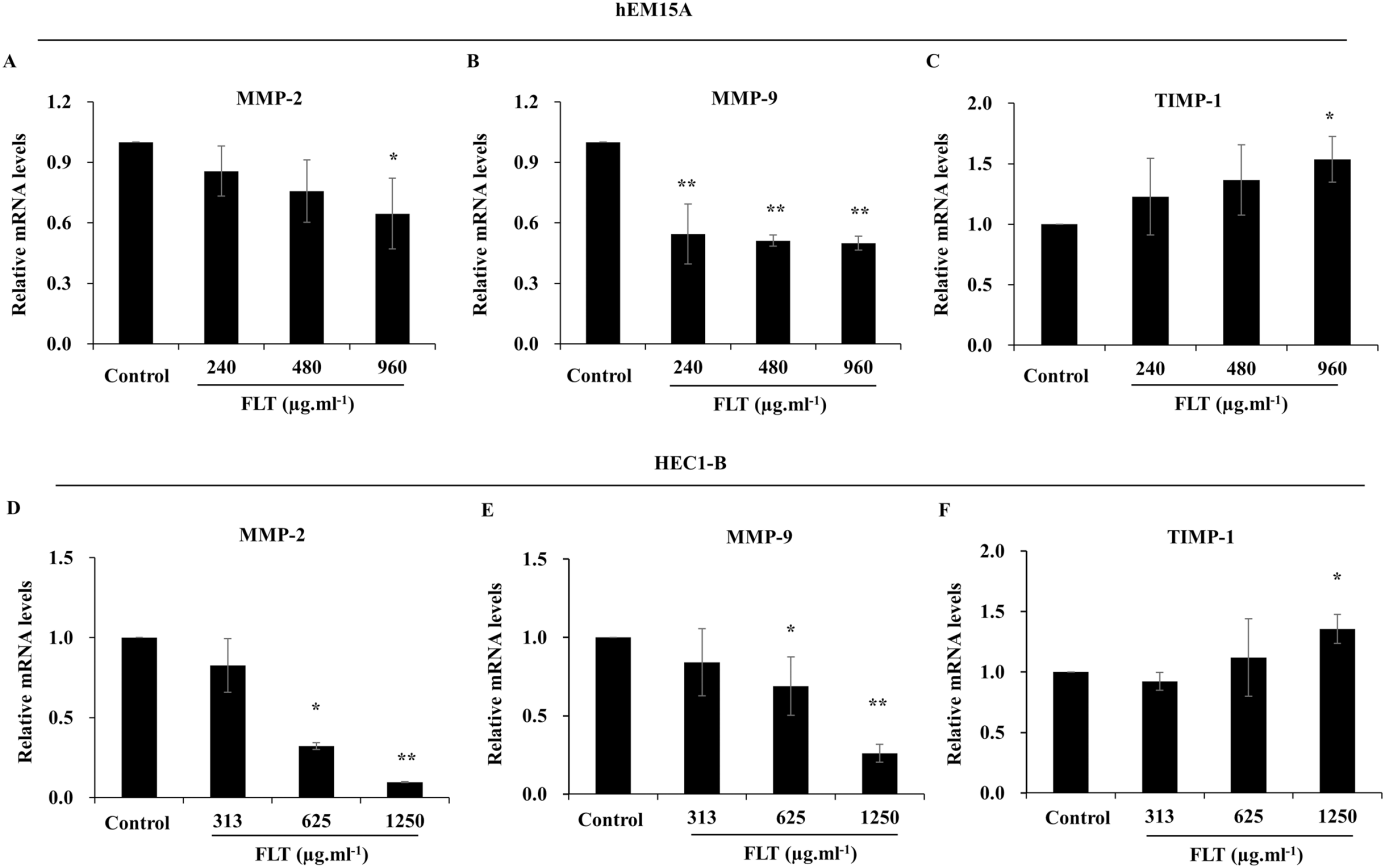
Gene expression of MMP/TIMP signaling adjusted by FLT for 24h. The mRNA levels of MMP-2, MMP-9, and TIMP-1 were detected by qPCR in hEM15A (A–C) and HEC1-B (D–F) cells. *P < 0.05 to control group, **P < 0.01 to control group. Columns, mean (*n* = 3). Bars, SD. FLT, ferulic acid, ligustrazine and tetrahydropalmatine.

After treated with FLT for 24 h, mRNA level of MMP-2 and MMP-9 obviously attenuated compared to the control group, while TIMP-1 expanded especially in 960 μg·ml^−1^ FLT group of hEM15A cells (*P* < 0.05) (Figs. 5A–5C). Moreover, mRNA level of MMP-2 and MMP-9 were significantly downregulated in HEC1-B cells. By contraries, TIMP-1 was upregulated in 1,250 μg·ml^−1^ FLT group (*P* < 0.05) (Figs. 5D–5F).

**Figure 6:**
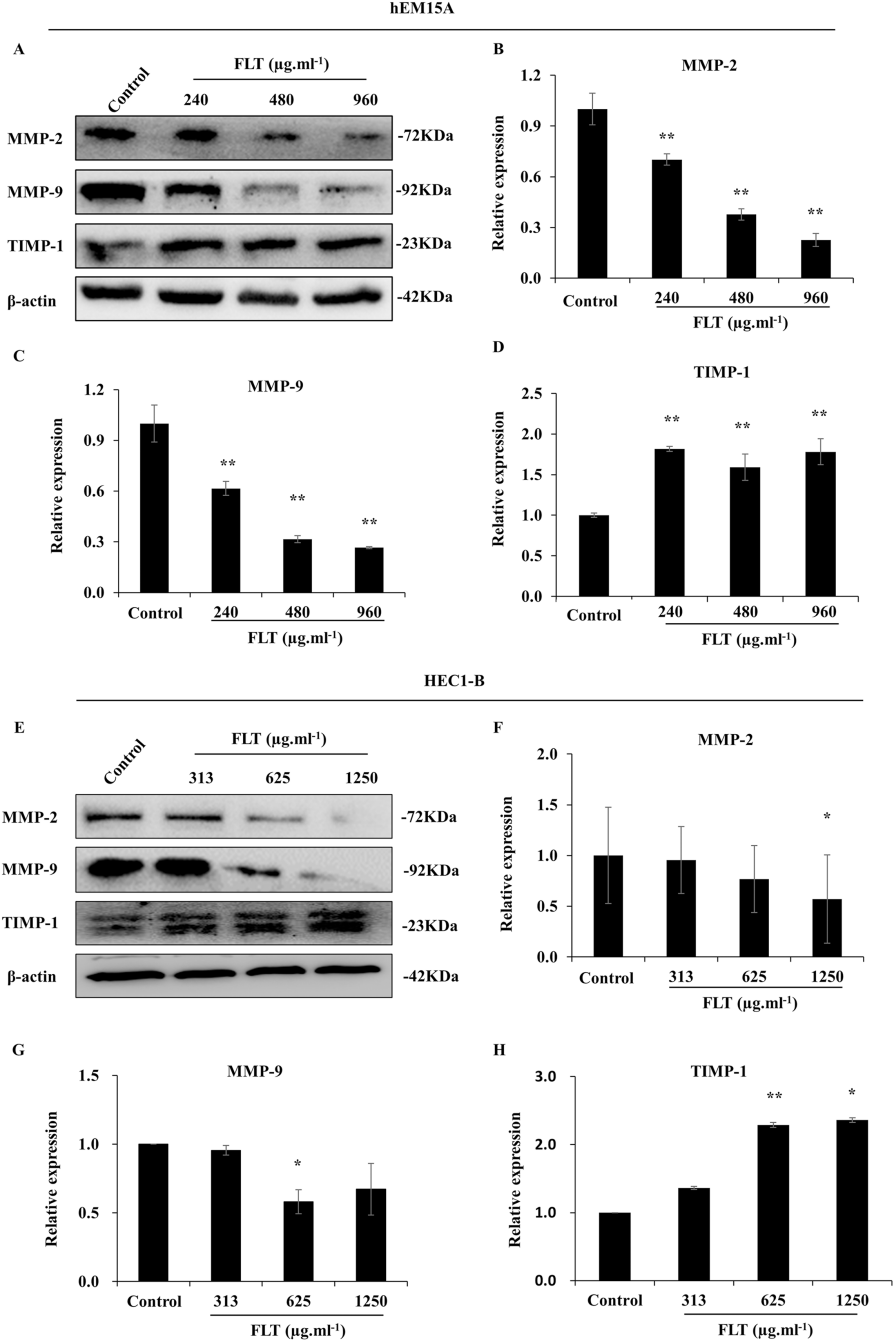
Modification of MMP/TIMP signaling protein treating with FLT for 24h. Protein levels of MMP-2, MMP-9, and TIMP-1 were detected by western blot in hEM15A (A–D) and HEC1-B (E–H) cells. The ratio of MMP-2, MMP-9, and TIMP-1 with β-actin were shown. *P < 0.05 to control group, **P < 0.01 to control group. Columns, mean (*n* = 3). Bars, SD. FLT, ferulic acid, ligustrazine and tetrahydropalmatine.

Furthermore, protein level was consistent with gene expression of MMP/TIMP signaling. MMP-2 and MMP-9 protein decreased in FLT groups, accompanied with TIMP-1 collection vs control group in hEM15A cells (*P* < 0.01) (Figs. 6A–6D). At the same time, MMP-2 protein in 1,250 μg·ml^−1^ FLT group, and MMP-9 in 625 μg·ml^−1^ FLT group were significantly downregulated. Though TIMP-1 were upregulated in 625 and 1,250 μg·ml^−1^ FLT groups compared with the control group in HEC1-B cells (*P* < 0.05) (Figs. 6E–6H).

## Discussion

FLT has displayed the potential anti-endometriosis effects in rat autograft EMS model (Chen et al., 2018; Tang et al., 2014; Wei et al., 2019). The underlying mechanism of FLT embrace diminishing the growth of EMS, suppression of E2, inflammation and epithelial mesenchymal transformation. Therefore, we detected the effects of FLT on endometrial cells and allograft EMS model. In this study, FLT markedly inhibited invasion and metastasis both in vitro and in vivo. It related to decrease of MMP-2, MMP-9, and increase of TIMP-1.

Nowadays, MMP/TIMP signaling is considered as one of vital mechanism in metastasis and invasion. MMPs is a kind of proteolytic enzymes. It can degrade extracellular matrix to supply the cell metastasis environment. On the contrary, TIMPs counteracts the degradation of MMPs (Herszenyi et al., 2012). In our experiment, invasion and metastasis were restrained by FLT, through suppressing MMP-2/9 and accumulating TIMP-1 in vivo and in vitro. Of note, gene expression and protein levels of MMP-2 and TIMP-1 were inconsistent in certain FLT doses. There might be two reasons. Firstly, FLT showed holistic effects of ferulic acid, ligustrazine, and tetrahydropalmatine. However, these three ingredients processed equivocal reaction on MMP/TIMP signaling. On one hand, metastatic suppression is found in three ingredients in various kinds of cancer, for example osteosarcoma, fibrosacroma, and breast cancer (Fang et al., 2018; Yodkeeree et al., 2013; Zhang et al., 2016). Especially, ferulic acid and ligustrazine show downregulation of MMP-2/9, and upregulation of TIMP-1 (Fahrioglu et al., 2016; Fang et al., 2013; Xu et al., 2018; Yue et al., 2019). In neuropathic pain or blood-brain barrier injury, ligustrazine or tetrahydropalmatine decreases the expression of MMP-2/9 (Jiang et al., 2017; Mao et al., 2015). On the other hand, using ligustrazine, bone marrow mesenchymal stem cells are promoted to migrate by raising MMP-2/9 (Wang et al., 2016). Secondly, certain concentration of FLT might influence MMP/TIMP protein through gene regulation or protein modification and degradation. It need further assessment.

Except MMP/TIMP signaling, there are still other mechanisms in metastasis and invasion. For example, fibrinolytic factors embrace fibrin degradation and then lead to metastasis. Urokinase plasminogen activator (uPA), plasminogen activator inhibitor (PAI)-1, and PAI-2 are the important fibrinolytic factors. uPA induce metastasis and invasion through activating plasminogen. PAI-1 and PAI-2 have the antagonistic effect with uPA (Andreasen et al., 1997; Liu et al., 2020). Only in one paper, ferulic acid expresses anti-invasion potential via decreasing uPA and increasing PAI-1, PAI-2 (Tsai et al., 2013). Ligustrazine and tetrahydropalmatine remain unexplored in uPA, PAI-1 and PAI-2. In this study, 313 μg·ml^−1^ FLT didn’t affect MMP-2/9 and TIMP-1 mRNA expression in HEC1B cells. But it decreased the invading cells. This discrepancy suggested FLT inhibited invasion not only through MMP/TIMP signaling. There might be another mechanism, such as uPA/PAI-1. Considering confusing data, it is worthwhile to explore role of FLT on fibrinolytic factors.

In cell viability assay, IC50 of FLT were different in hEM15A and HEC1-B cells, which were human endometriosis-derived eutopic endometrium stromal cells and endometrial adenocarcinoma cells. It was also notable that IC50 in HEC1-B cells were obviously lower than that in hEM15A cells. It was indicated that HEC1-B cells might be more sensitive to FLT. There are no researches of FLT or FLT ingredients, ferulic acid, ligustrazine, or tetrahydropalmatine on endometrial cancer, except *Angelica sinensis* showed weak binding with ER in endometrial cancer cells (Liu et al., 2001). Therefore, our results are suggested that FLT or FLT ingredients might have the potential effects on endometrial cancer. It needs further research.

Gestrinone is an androgen derived from 19-nortestosterone steroid, which has been widely applied in clinical treatment of EMS. However, its adverse effects include weight gain, headache, abnormal cholesterol, nausea, and hirsutism (Vercellini et al., 2009). Invasion and metastasis of endometrial implantation is essential pathological step in EMS (Chui, Wang & Shih, 2017; Mihailovici et al., 2017). Until now, gestrinone has not been shown to be effective for invasion and metastasis. Here, FLT performed the inhibition of invasion and metastasis in EMS. This data suggested that FLT might be the potential treatment as an alternative or combination treatment modality.

## Conclusions

FLT prevented cell viability, invasion and metastasis in endometrial hEM15A and HEC1-B cells, meanwhile reduced volume of ectopic endometrium in allograft EMS. It is related to the regulation of MMP/TIMP signaling by FLT, through attenuating MMP-2 and MMP-9, accumulating TIMP-1 both in vitro and in vivo. These results provide a fundamental basis for the pharmacological application of FLT in the potential treatment of EMS as an alternative or combination treatment modality.

## Supplemental Information

10.7717/peerj.11664/supp-1 Supplemental Information 1 Author checklist.

10.7717/peerj.11664/supp-2 Supplemental Information 2 Western blot image of Fig 1.

10.7717/peerj.11664/supp-3 Supplemental Information 3 Western blot images of Fig 6.

10.7717/peerj.11664/supp-4 Supplemental Information 4 Raw data of all figures.

## Additional Information and Declarations

### Data Availability

The following information was supplied regarding data availability: The western blot results are available in the Supplemental Files.

## References

[ref-1] Andreasen PA, Kjoller L, Christensen L, Duffy MJ (1997). The urokinase-type plasminogen activator system in cancer metastasis: a review. International Journal of Cancer.

[ref-2] Attaman JA, Stanic AK, Kim M, Lynch MP, Rueda BR, Styer AK (2014). The anti-inflammatory impact of omega-3 polyunsaturated Fatty acids during the establishment of endometriosis-like lesions. American Journal of Reproductive Immunology.

[ref-3] Bai H, Li PQ, Liu J, Liu XP (2014). Association analysis on traditional efficacy and modern research of Foshou San. Chinese Traditional Patent Medicine.

[ref-5] Chen Y, Wei J, Zhang Y, Sun W, Li Z, Wang Q, Xu X, Li C, Li P (2018). Anti-endometriosis mechanism of jiawei foshou san based on network pharmacology. Frontiers in Pharmacology.

[ref-6] Cheng CW, Licence D, Cook E, Luo F, Arends MJ, Smith SK, Print CG, Charnock-Jones DS (2011). Activation of mutated K-ras in donor endometrial epithelium and stroma promotes lesion growth in an intact immunocompetent murine model of endometriosis. Journal of Pathology.

[ref-7] Chui MH, Wang TL, Shih IM (2017). Endometriosis: benign, malignant, or something in between?. Oncotarget.

[ref-9] Fahrioglu U, Dodurga Y, Elmas L, Secme M (2016). Ferulic acid decreases cell viability and colony formation while inhibiting migration of MIA PaCa-2 human pancreatic cancer cells in vitro. Gene.

[ref-10] Fang M, Mei X, Yao H, Zhang T, Zhang T, Lu N, Liu Y, Xu W, Wan C (2018). Beta-elemene enhances anticancer and anti-metastatic effects of osteosarcoma of ligustrazine in vitro and in vivo. Oncology Letters.

[ref-11] Fang HY, Wang HM, Chang KF, Hu HT, Hwang LJ, Fu TF, Lin YC, Chang WC, Chiu TP, Wen ZH, Fong Y, Chiu CC, Chen BH (2013). Feruloyl-L-arabinose attenuates migration, invasion and production of reactive oxygen species in H1299 lung cancer cells. Food and Chemical Toxicology.

[ref-12] Ferrero S, Evangelisti G, Barra F (2018). Current and emerging treatment options for endometriosis. Expert Opinion on Pharmacotherapy.

[ref-13] Herszenyi L, Hritz I, Lakatos G, Varga MZ, Tulassay Z (2012). The behavior of matrix metalloproteinases and their inhibitors in colorectal cancer. International Journal of Molecular Sciences.

[ref-15] Jiang L, Pan CL, Wang CY, Liu BQ, Han Y, Hu L, Liu L, Yang Y, Qu JW, Liu WT (2017). Selective suppression of the JNK-MMP2/9 signal pathway by tetramethylpyrazine attenuates neuropathic pain in rats. Journal of Neuroinflammation.

[ref-16] Liao F (2000). Herbs of activating blood circulation to remove blood stasis. Clinical Hemorheology and Microcirculation.

[ref-17] Liu JH, Burdette JE, Xu HY, Gu CG, van Breemen RB, Bhat KPL, Booth N, Constantinou AI, Pezzuto JM, Fong HHS, Farnsworth NR, Bolton JL (2001). Evaluation of estrogenic activity of plant extracts for the potential treatment of menopausal symptoms. Journal of Agricultural and Food Chemistry.

[ref-18] Liu J, Chen Z, Huang M, Tang S, Wang Q, Hu P, Gupta P, Ashby CR, Chen ZS, Zhang L (2020). Plasminogen activator inhibitor (PAI) trap3, an exocellular peptide inhibitor of PAI-1, attenuates the rearrangement of F-actin and migration of cancer cells. Experimental Cell Research.

[ref-19] Mao XW, Pan CS, Huang P, Liu YY, Wang CS, Yan L, Hu BH, Chang X, He K, Mu HN, Li Q, Sun K, Fan JY, Han JY (2015). Levo-tetrahydropalmatine attenuates mouse blood-brain barrier injury induced by focal cerebral ischemia and reperfusion: involvement of Src kinase. Scientific Reports.

[ref-20] Mihailovici A, Rottenstreich M, Kovel S, Wassermann I, Smorgick N, Vaknin Z (2017). Endometriosis-associated malignant transformation in abdominal surgical scar: a PRISMA-compliant systematic review. Medicine (Baltimore).

[ref-21] Tang Q, Shang FH, Wang XC, Yang Y, Chen G, Chen Y, Zhang JF, Xu XY (2014). Combination use of ferulic acid, ligustrazine and tetrahydropalmatine inhibits the growth of ectopic endometrial tissue: a multi-target therapy for endometriosis rats. Journal of Ethnopharmacology.

[ref-22] Tsai CM, Yen GC, Sun FM, Yang SF, Weng CJ (2013). Assessment of the anti-invasion potential and mechanism of select cinnamic acid derivatives on human lung adenocarcinoma cells. Molecular Pharmaceutics.

[ref-23] Vercellini P, Somigliana E, Vigano P, Abbiati A, Barbara G, Crosignani PG (2009). Endometriosis: current therapies and new pharmacological developments. Drugs.

[ref-24] Wang J, Qu TB, Chu LS, Li L, Ren CC, Sun SQ, Fang Y (2016). Ligustrazine promoted the migration of bone marrow mesenchymal stem cells by up-regulating MMP-2 and MMP-9 expressions. Zhongguo Zhong Xi Yi Jie He Za Zhi.

[ref-25] Wei JH, Zhao BX, Zhang CL, Shen BB, Zhang Y, Li CX, Chen Y (2019). Jiawei Foshou San induces apoptosis in Ectopic endometrium based on systems pharmacology, molecular docking, and experimental evidence. Evidence-Based Complementary and Alternative Medicine.

[ref-26] Xu D, Chi G, Zhao C, Li D (2018). Ligustrazine inhibits growth, migration and invasion of medulloblastoma daoy cells by up-regulation of miR-211. Cellular Physiology and Biochemistry.

[ref-27] Yodkeeree S, Wongsirisin P, Pompimon W, Limtrakul P (2013). Anti-invasion effect of crebanine and O-methylbulbocapnine from Stephania venosa via down-regulated matrix metalloproteinases and urokinase plasminogen activator. Chemical and Pharmaceutical Bulletin.

[ref-28] Yue SJ, Zhang PX, Zhu Y, Li NG, Chen YY, Li JJ, Zhang S, Jin RY, Yan H, Shi XQ, Tang YP, Duan JA (2019). A ferulic acid derivative FXS-3 inhibits proliferation and metastasis of human lung cancer A549 cells via positive JNK signaling pathway and negative ERK/p38, AKT/mTOR and MEK/ERK signaling pathways. Molecules.

[ref-29] Zhang X, Lin D, Jiang R, Li H, Wan J, Li H (2016). Ferulic acid exerts antitumor activity and inhibits metastasis in breast cancer cells by regulating epithelial to mesenchymal transition. Oncology Reports.

