# Supplementary figures and images for: Combination of ferulic acid, ligustrazine and tetrahydropalmatine inhibits invasion and metastasis through MMP/TIMP signaling in endometriosis
Source: PeerJ. 2021 Jun 28;9:e11664. doi: 10.7717/peerj.11664 (PMC8247703; doi:10.7717/peerj.11664)

Figure 1

E

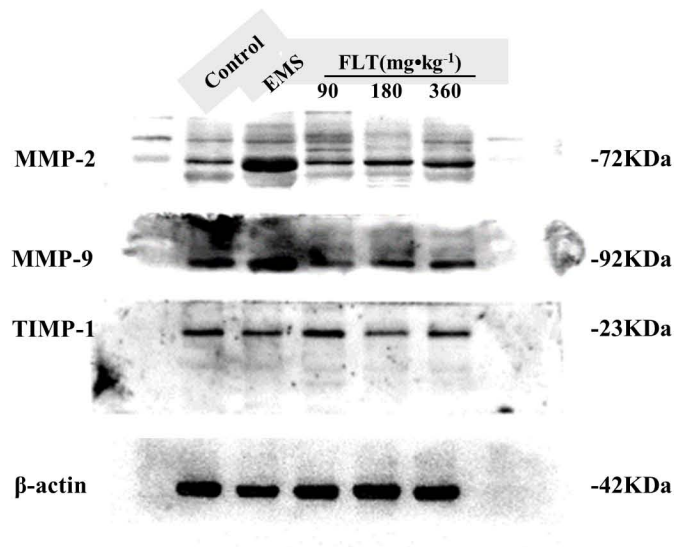

Supplement: Supplemental Information 2 [file peerj-09-11664-s002.pdf]

Figure 6

A

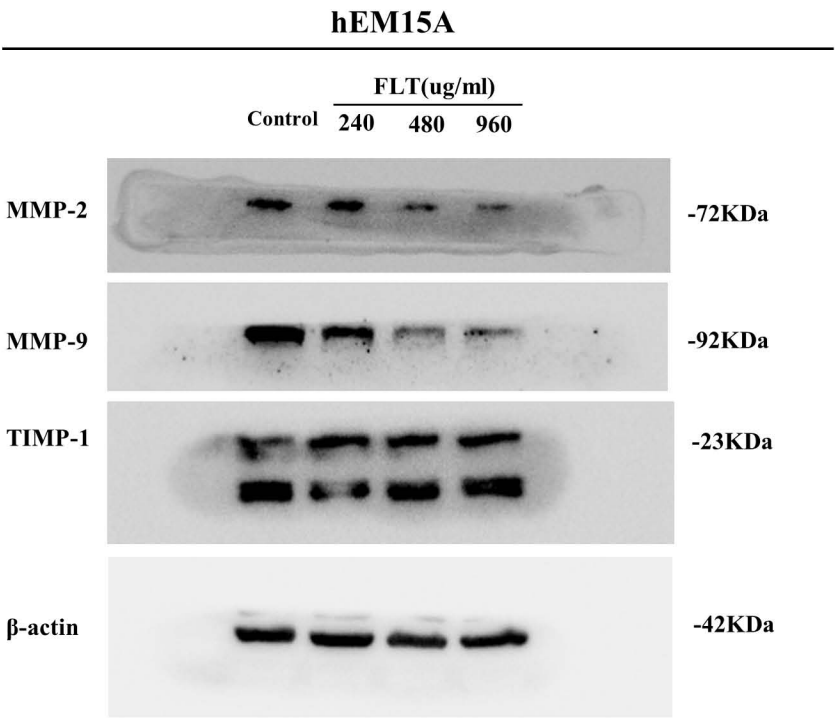

E

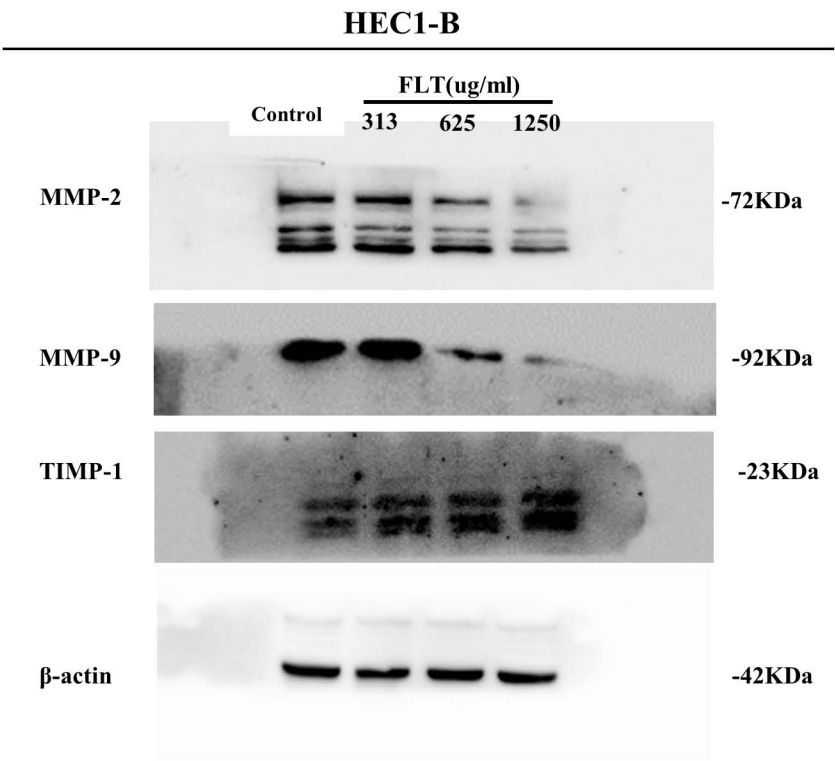

Supplement: Supplemental Information 3 [file peerj-09-11664-s003.pdf]
